# Socio-demographic correlates of six indicators of alcohol consumption: survey findings of students across seven universities in England, Wales and Northern Ireland

**DOI:** 10.1186/2049-3258-71-29

**Published:** 2013-11-06

**Authors:** Walid El Ansari, Rene Sebena, Christiane Stock

**Affiliations:** 1 Department of Sport and Exercise, Faculty of Applied Sciences, University of Gloucestershire, Oxstalls Campus, Oxstalls Lane, Gloucester GL2 9HW, UK; 2 Department of Psychology, Faculty of Arts, PJ Safarik University, Kosice, Slovak Republic; 3 Unit for Health Promotion Research, University of Southern Denmark, Esbjerg, Denmark

**Keywords:** Heavy episodic drinking, Problem drinking, Alcohol dependence, University students, Sociodemographic and educational characteristics, Gender

## Abstract

**Background:**

This study assessed the prevalence of six alcohol consumption indicators in a sample of university students. We also examined whether students’ sociodemographic and educational characteristics were associated with any of the six alcohol consumption indicators; and whether associations between students’ sociodemographic and educational characteristics and the six alcohol consumption indicators differed by gender.

**Methods:**

A cross-sectional study of 3706 students enrolled at 7 universities in England, Wales and Northern Ireland. A self-administered questionnaire assessed six alcohol consumption measures: length of time of last (most recent) drinking occasion; amount consumed during last drinking occasion; frequency of alcohol consumption; heavy episodic drinking (≥ 5 drinks in a row); problem drinking; and possible alcohol dependence as measured by CAGE. The questionnaire also collected information on seven relevant student sociodemographic characteristics (age, gender, academic year of study, current living circumstances - accommodation with parents, whether student was in intimate relationship, socioeconomic status of parents - parental education, income sufficiency) and two academic achievement variables (importance of achieving good grades at university, and one’s academic performance in comparison with one’s peers).

**Results:**

The majority of students (65% of females, 76% of males) reported heavy episodic drinking at least once within the last 2 weeks, and problem drinking was prevalent in 20% of females and 29% of males. Factors consistently positively associated with all six indicators of alcohol consumption were male gender and perceived insufficient income. Other factors such as living away from home, being in 1^st^ or 2^nd^ year of studies, having no intimate partner, and lower academic achievement were associated with some, but not all indicators of alcohol consumption.

**Conclusions:**

The high level of alcohol consumption calls for regular/periodic monitoring of student use of alcohol, and for urgent preventive actions and intervention programmes at the universities in the UK.

## Background

Drinking alcohol in the period of college and university study is a social challenge that warrants research attention, and the consequences and implications of binge and hazardous drinking among young people including university students comprise a challenge of shared international concern. College and university students in many countries are at increased risk for heavy drinking, with serious immediate health risks (e.g. drink-driving and other substance use), and longer-term risks (e.g. alcohol dependence) [1]. Certainly, alcohol consumption of college students has impact on the students themselves and also the college community in general, where the misuse of alcohol can lead to a wide variety of consequences, the most severe being alcohol abuse, dependence, and death [2].

For instance, in New Zealand, hazardous drinking was widespread and persistent among tertiary students living in the halls of residence, where the 60% and 58.2% of male and female drinkers respectively typically consumed more than the national safe drinking guidelines [3]. Across undergraduates in Nigeria, prevalence of alcohol use was 40.6%, and heavy episodic alcohol use was reported by 31.1% using the AUDIT questionnaire [4]. Similarly, across students enrolled at four universities in Slovakia, 41% of students drank alcohol ≥ 1 time a week, 77% reported heavy episodic drinking, 49% had been drunk more than once in the last month, and problem drinking existed in 23.3% of the sample [5]. Indeed, a web-based survey in New Zealand (2548 undergraduates) found that 81% of both women and men drank in the previous 4 weeks, 37% reported ≥ 1 binge episodes in the last week, 14% (women) and 15% (men) had ≥ 2 binge episodes in the last week, and 68% scored in the hazardous range (4+) on the AUDIT consumption subscale [3]. Likewise, in Brazil, a survey of 608 university students showed a prevalence of alcohol abuse of 18.3% in men and 6.1% in women [6]. Such findings emphasize the vulnerability of these young adult university student populations to risky health behaviors, particularly that a range of socioeconomic characteristics seems to be associated with various patterns of alcohol consumption, albeit findings are not always clear-cut.

### Gender

Among undergraduates of a Nigerian tertiary institution, male gender was associated with problem drinking [4]. In Brazil, heavier alcohol consumption and alcohol abuse were observed in male university students [6]. Indeed male students were more likely to use alcohol, and among university student drinkers in Croatia, males consumed alcohol at a higher frequency than females [7]. Likewise, across several European countries, male students had higher scores on the CAGE screening instrument for alcohol dependence [8,9]. Conversely, studies in northern Europe assessed the prevalence of alcohol consumption to report an absence of gender differences e.g. [10]. Similarly, in the Netherlands [11], no significant correlations were found between alcohol use and gender across a sample of Dutch dental students.

### Age and academic year of study

Across undergraduates in southwestern Nigeria, older age was associated with problem drinking [4]. Likewsie, in Brazil, heavier alcohol consumption and alcohol abuse were observed in older students [6]. Conversely, research in Slovakia reported that a higher study year was associated only with lower levels of heavy episodic drinking, and displayed no association with the other variables that were examined (frequency of alcohol use, frequency of drunkenness, and problem drinking) [5].

Certainly, the pattern of changes in alcohol consumption over the academic years differs across different studies. Some studies reported decreases over the academic years e.g. [12], whilst other researchers found a peak in the middle years [13]. In contrast, studies in other European and Scandinavian countries showed no difference at all in alcohol consumption over the academic years [14,15]. Furthermore, research elsewhere found differing trends for male and female students [15]. Likewise, in the Netherlands [11] there were no significant correlations between alcohol use and years in dental education across a sample of Dutch dental students.

### Socioeconomic status

In a survey of 443 students at a university in Nigeria, higher paternal education was associated with problem drinking [4]. The subjectively evaluated economic status of students’ families was related to regular alcohol use. In Sweden, students with a higher disposable income were more likely to engage in risky single occasion drinking [14], as were students (in 21 mostly European countries) who designated themselves as belonging to the wealthier 50% of the population [16]. Conversely, in Slovakia, higher parental education was associated only with lower levels of problem drinking, but displayed no association with the other variables that were scrutinized (frequency of alcohol use, frequency of drunkenness, and heavy episodic drinking) [5].

### Current living circumstances

A recent review [17] found that current living circumstances of students were associated with alcohol use: students living in situations characterized by less control (e.g., living alone, with roommates, in student halls) and without family obligations (i.e., not living with their parent, their partner or their children) were more likely to use alcohol more frequently, in higher quantities, and engage in risky single occasion drinking more often. The report suggested that in Nordic countries, students with more family obligations (e.g. in a serious relationship or with children) were less likely to consume high volumes of alcohol and to engage in risky single occasion drinking. In agreement, in Slovakia, living with parents during the semester was consistently associated with less frequent heavy episodic drinking, drunkenness episodes, and problem drinking, while having an intimate relationship was associated with less problem drinking only [5].

### Academic achievement

A majority of research demonstrated that alcohol use and especially misuse is negatively associated with indicators of academic achievement. A review summarized the consequences of alcohol misuse on college campus and concluded that “a substantial amount of empirical research is available demonstrating a connection between alcohol consumption and impaired academic performance” [18]. Heavy episodic drinkers have also been shown to be more likely than their non binge drinking peers to report that drinking caused them to miss class, fall behind in their schoolwork, and perform poorly on test/s or other academic project/s [19]. A significant negative association was also found between semester academic performance and objectively measured alcohol indicators related to breath alcohol concentration [20].

Generally three points stand out. The first is that measures of alcohol-related problems for college students need to assess specific dimensions pertaining to 3 main domains: alcohol abuse, alcohol dependence, and risky drinking [2]. Hence, research needs to consider such multiple aspects of alcohol use to include information about both intensity and frequency. Fluctuations in alcohol use are marked among young adults, and acute consequences could be affected more by intensity than frequency of alcohol use [21]. Hence ideally inquiries need to include: a) frequency of alcohol consumption; b) volume or “level of drinking”, (average amount of alcohol consumed per week in grams); c) risky single occasion drinking or drinking to intoxication (often measured by questions such as “how often do you have six or more drinks on one occasion?”); d) indicators for alcohol use disorder or alcohol dependence based on screening instruments, e.g. CAGE [22] etc. [17]. In spite of such proposals advocating the measurement of the frequency, quantity and volume of alcohol consumption, frequently, published studies have traditionally examined a much narrower 'spectrum’ of alcohol use of university students. For instance, some studies focused only on measure/s of volume e.g. [12] or frequency [23], whilst others focused mainly on risky single occasion drinking [16]. Likewise in the UK, researchers [24] reported on two aspects (binge drinking - having had ≥5 drinks in a row in last two weeks, and problem drinking). In Slovakia, research across 4 universities investigated only four aspects (frequency of alcohol use, heavy episodic drinking, frequency of drunkenness and problem drinking) [5].

The second feature is that a range of sociodemographic characteristics seems associated with different patterns of alcohol consumption, although studies in many instances show inconsistent findings. The third point is that whilst many studies gathered information on students’ alcohol use employing data from one university per country e.g. [4,6]; fewer studies collected data from > 1 university per country - four universities e.g. [5]; five universities e.g. [3]; and indeed much less research endeavored to collect data from a larger number of universities. There are very few notable exceptions.

Hence few studies examined the associations between a wider range of sociodemographic characteristics and wider range of different measures of alcohol consumption of undergraduate student populations across several universities, whilst simultaneously considering the potential roles of academic achievement variables which are important variables related to alcohol consumption; and also whilst controlling for a range of demographic variables (e.g. age, gender, having intimate partner, accommodation with parents during the semester). The study described in this paper bridges these gaps in knowledge to contribute to the evidence base.

### Aim of the study

The current research assessed the associations between students’: a) sociodemographic characteristics (age, gender, year of study at university, type of accommodation, being in an intimate relationship), socioeconomic status (parental education and income sufficiency), and b) academic (educational achievement) characteristics (importance of good grades, and performance relative to peers) on the one hand; and c) six indicators of alcohol consumption [Length of time of the last (most recent) drinking occasion, amount (number of drinks) of alcohol consumed during the last (most recent) drinking occasion, high frequency of drinking, frequency of heavy episodic drinking, problem drinking, and possible alcohol dependence] on the other. The study assessed gender differences and university differences of students enrolled at seven universities in England, Wales and Northern Ireland. The three specific objectives were to determine:

• the prevalence of alcohol use related to six alcohol consumption indicators;

• whether (and which) students’ sociodemographic and educational characteristics were associated with any of the six alcohol consumption indicators; and,

• whether associations between students’ sociodemographic and educational characteristics and the six alcohol consumption indicators differed by gender.

## Methods

### Sample, ethics and data collection procedures

The research ethics committees at the participating universities provided ethical approval for the study. After permission from the course/module tutors, students were provided with self-administered questionnaires to complete during the last 10 minutes of lectures that they were attending. Each questionnaire had a participant information sheet outlining the study aims/objectives. Participation was voluntary and anonymous, no monetary or course credit incentives were provided to participants, and data were confidential and protected. Students were informed that by completing the questionnaire, they agreed to take part in the study. Data was collected in 2007–2008 at the seven participating universities in England, Wales and Northern Ireland. A representative sample of students was sought at all participating universities, and all data were computer-entered at one central site in order to maximize quality assurance and minimize data entry errors.

Data used in the current analysis was collected as part of a general Student Health Survey implemented in many European and African countries [25-32]. We employed data from 3,706 undergraduate students at seven universities in England (University of Gloucestershire, Bath Spa University, Oxford Brookes University, University of Chester, Plymouth University); Wales (Swansea University); and Northern Ireland (University of Ulster). Based on the number of completed returned questionnaires, the response rate was about 80%. Higher proportions of Year 1 students were represented at 3 universities (Chester, Bath Spa, Swansea), while for the rest of the sample Year 2 participants contributed slightly more data, with the exception of Plymouth where it was the Year 3 students. In this article, we employ the terms 'university’ and 'college’ interchangeably to denote higher education institutions, and the average age of entry to university in the UK is about 18 years.

### Measures

#### ***Length of time of the last (most recent) drinking occasion (1 item)***

“The last time you 'partied’/socialized, how many hours did you drink alcohol?” Participants provided the number of hours. As the median and mean were almost the same, (Mean = 5.3 hours, Median = 5), thus, using mean split the number of hours was then dichotomized into 'High’ and 'Low’ length of time of drinking.

#### ***Amount (number of drinks) of alcohol consumed during the last (most recent) drinking occasion (1 item)***

“The last time you 'partied’/socialized, how many alcoholic drinks did you have? (including alcoholic drinks you possibly had before going out)”. Participants provided the number of drinks. A “drink” is defined a glass of wine (ca 15 cl), a bottle or can of beer (ca 50 cl), a shot glass of spirits (ca 5 cl) or a mixed drink.) As the median and mean were almost the same (Mean = 7.6 drinks, Median = 7 drinks), thus, using mean split, the number of drinks was then dichotomized into 'High’ and 'Low’ amount of drinking.

#### ***Frequency of alcohol consumption (1 item)***

Measured using the question “Over the past three months how often have you drunk alcohol, for example, beer?” (response options: “never,” “once a week or less,” “once a week,” “a few times each week,” “every day,” “a few times each day”, later dichotomised into Low frequency = “drinking once a week or less” versus High frequency = “drinking a few times or more each week”.

#### ***Frequency of heavy episodic drinking (1 item)***

“Think back over the last two weeks. How many times, if any, have you had five or more alcoholic drinks at a sitting?” Respondents were classified into non-episodic drinkers (if they responded “never”) and heavy episodic drinkers (all others).

#### ***Problem drinking (4 items)***

We included a brief alcoholism-screening test, the CAGE test [22] comprising four questions (Have you ever felt you should Cut down on your drinking? Have people Annoyed you by criticizing your drinking? Have you ever-felt bad or Guilty about your drinking? Have you ever had a drink in the morning to get rid of a hangover? (Eye opener). Each question is answered either “yes” or “no.” Two or more affirmative answers suggested problem drinking. We categorized respondents as non-problem drinkers (< 2 positive responses) and problem drinkers (≥ 2 positive responses).

#### ***Possible alcohol dependence (4 items)***

CAGE test was also used to identify possible alcohol dependence. It has been proposed that three and more positive responses in CAGE seriously suggested alcohol dependence. We categorized respondents as not possible alcohol dependence (< 3 positive responses) and possible alcohol dependence (≥ 3 positive responses).

#### ***Sociodemographic variables (7 items)***

Age, gender and year of study at university were based on individuals’ self-reported responses in the questionnaire. Students were also asked about the type of accommodation they lived in during the semester, and responses were dichotomized into “I live with my parents” versus “I do not live with my parents.” In addition, participants were asked whether they currently were in an intimate relationship (i.e. having a partner).

#### ***Socioeconomic status (SES) (2 items)***

Determined by employing two separate indicators. The first SES indicator was *parental education*. This comprised the level of education of both parents of each student: the questionnaire inquired about father’s and mother’s educational status individually – “What is the highest education level of your mother, father?” (response options: “No formal education,” “Secondary vocational school,” “A levels,” “Bachelor’s degree,” “Master’s degree and Ph.D. or equivalent”). For the current analysis, each parents’ educational levels were collapsed into two categories (Low - A levels and lower degree; and High - bachelor’s degree and above). Then, father’s and mother’s educational status (Low vs. High) were combined to generate four groups: both parents high, both parents low, mother high/father low, and father high/mother low.

#### ***Income sufficiency (1 item)***

“How sufficient do you consider your income?” with four Likert scale responses (“always sufficient,” “mostly sufficient,” “mostly insufficient” or “insufficient”) which were then dichotomized into “always sufficient” versus “other.”

#### ***Educational (academic achievement) variables (2 items)***

The current study conceptualized and measured academic performance using 2 approaches [25]: (1) students’ *internal reflection* on their academic achievement in terms of the importance they attach to achieving good grades in their studies “How important is it for you to have good grades at university?” (4 response categories, 1 = 'very important’, 2 = somewhat important’, 3 = 'not very important’, 4 = 'not at all important’). We dichotomized this variable into 1 = 'very important’ versus 2 = 'other’; and, (2) as students’ *subjective comparative appraisal* of their overall academic performance in comparison with their peers - “How do you rate your performance in comparison with your fellow students?” (5 response categories, 1 = 'much better’, 2 = 'better’, 3 = 'same’, 4 = 'worse’, 5 = 'much worse’). We dichotomized this variable into 1, 2, 3 =1 versus 4, 5 =2).

### Statistical analysis

All statistical analyses were computed using the package SPSS v20, with significance level set at P <0.05. Descriptive analysis of alcohol consumption, problem drinking and other studied variables was undertaken separately for each university. Differences in frequencies between universities were computed using Chi-square Test (χ2) and ANOVA. In the next step, the associations between sociodemographic and educational variables on the one side and all the drinking patterns on the other were analyzed in logistic regression models. Additionally, all two-way gender interactions were assessed. Only significant interactions were reported and included in the final tables. Results are reported as odds ratios (OR) and 95% confidence intervals (CI). Listwise deletion was undertaken for handling the missing data.

## Results

### Characteristics of the sample

The sample (N = 3706) comprised 970 students from the University of Gloucestershire (43.6%; *M* age 23.4 years ± 8.4 SD); 429 students from Bath Spa University (22.6%; *M* age 22.2 ± 7.0 SD); 208 students from Oxford Brookes University (10.8%; *M* age 31.6 ± 10.5 SD); 993 students from the University of Chester (13.1%; *M* age 26 ± 9.2 SD); 169 students from Plymouth University (56.2%; *M* age 24.6 ± 7.2 SD); 406 students from Swansea University (7.8%; *M* age 25.0 ± 7.4 SD); and 475 students from the University of Ulster (8.2%; *M* age 25.2 ± 7.7 SD).

Selected characteristics of the study population are depicted in Table 1. Across the seven universities, the majority of participants were females, as females were more represented at most universities, possibly due to the nature of the schools (e.g., Schools of Nursing, of Health Sciences, or of Health & Social Care, etc.) at each university where the data were collected. Differences in gender composition were less pronounced in the Gloucestershire sample. However such gender composition of the student body is in line with the latest statistics released by the University and Colleges Admissions Service (Ucas) that showed a 22,000 drop in the number of male students enrolling at university. This meant that across the autumn of 2012, women were a third more likely to start a degree than their male counterparts, despite the fact that there are actually more young men than women in the UK [33].

**Table 1:** Selected characteristics of the survey by participating universities in the United Kingdom (Academic year 2007–2008)

	**Whole sample**	**Chester**	**Gloucestershire**	**Ulster**	**Swansea**	**Plymouth**	**Oxford Brookes**	**Bath spa**	**p**
	**N = 3706**	**N = 993**	**N = 970**	**N = 475**	**N = 406**	**N = 169**	**N = 208**	**N = 429**	
**Sociodemographic variables**	**N (%)**	**N (%)**	**N (%)**	**N (%)**	**N (%)**	**N (%)**	**N (%)**	**N (%)**	
Gender (Female)	2699 (77.9)	757 (86.9)	512 (56.4)	425 (91.8)	367 (92.2)	108 (63.9)	174 (89.2)	356 (77.4)	<0.001
Age [year (SD)]	24.9 (8.6)	26 (9.2)	23.4 (8.4)	25.2 (7.7)	25 (7.4)	24.6 (7.2)	31.6 (10.5)	22.2 (7)	<0.001
Year of study									<0.001
1st year	1491 (42.6)	552 (61.6)	311 (34.5)	104 (22.5)	190 (47.7)	32 (18.9)	45 (22.4)	257 (54.1)	
2nd year	1095 (31.3)	200 (22.3)	330 (36.6)	204 (44.2)	94 (23.6)	59 (34.9)	97 (48.3)	111 (23.4)	
3rd year	655 (18.7)	74 (8.3)	157 (17.4)	151 (32.7)	88 (22.1)	73 (43.2)	6 (3)	106 (22.3)	
≥ 4th year	262 (1.3)	70 (7.8)	104 (11.5)	3 (0.6)	26 (6.5)	5 (3)	53 (26.4)	1 (0.2)	
Have intimate partner	1748 (55.8)	429 (55.7)	438 (52.2)	251 (62.3)	211 (58.9)	101 (63.1)	83 (52.9)	235 (53.0)	<0.01
Accommodation with parents	890 (24)	243 (24.5)	147 (15.2)	203 (42.7)	113 (27.8)	22 (13)	42 (20.2)	120 (24.7)	<0.001
Parental education									<0.001
Both parents low	2448 (78.4)	542 (70.6)	595 (74)	340 (80)	309 (87.3)	130 (83.9)	151 (83.9)	381 (87.2)	
Mother high, father low	209 (6.7)	78 (10.2)	53 (6.6)	43 (10.1)	15 (4.2)	1 (0.6)	4 (2.2)	15 (3.4)	
Father high, mother low	281 (9.0)	87 (11.3)	89 (11.1)	20 (4.7)	23 (6.5)	18 (11.6)	18 (10.0)	26 (5.9)	
Both parents high	185 (5.9)	61 (7.9)	67 (8.3)	22 (5.2)	7 (2.0)	6 (3.9)	7 (3.9)	15 (3.4)	
Perceived income sufficiency*	1423 (42.5)	314 (35.7)	430 (51.9)	111 (258)	147 (37.2)	73 (44.8)	106 (53.8)	242 (53.3)	<0.001
**Alcohol consumption variables**									
High length of time of drinking**	1271 (37.9)	363 (40.1)	352 (40.6)	146 (33.6)	164 (44.0)	49 (30.1)	44 (25.1)	153 (35.3)	<0.001
High amount of drinking***	1535 (46.5)	414 (47.0)	466 (54.4)	195 (45.1)	178 (48.5)	60 (37.5)	48 (26.8)	174 (40.6)	<0.001
High frequency of drinking	1508 (42.7)	341 (38)	525 (56.6)	129 (27.7)	145 (36.3)	67 (39.9)	89 (43.6)	212 (45.2)	<0.001
Heavy episodic drinking	2136 (67.2)	548 (65.6)	612 (74.7)	291 (69.8)	232 (65)	94 (59.1)	81 (47.9)	278 (66)	<0.001
Problem drinking	714 (22.4)	162 (19.8)	204 (24.3)	122 (28.8)	52 (13.5)	21 (25.9)	39 (20.5)	114 (25.4)	<0.001
Possible alcohol dependence	279 (8.8)	54 (6.6)	88 (10.5)	45 (10.6)	20 (5.2)	6 (7.4)	15 (7.9)	51 (11.4)	<0.01
**Educational (academic achievement) variables**									
High importance to achieve good grades	2204 (63.3)	549 (62.7)	575 (62.8)	330 (71.3)	237 (59.4)	107 (64.5)	113 (56.8)	293 (63.4)	<0.01
Academic performance compared to peers (Same or better)	2831 (82.0)	679 (79.0)	754 (83.2)	368 (79.7)	331 (82.8)	140 (83.8)	167 (85.6)	392 (84.5)	.057

*Always sufficient; **High length of time of last (most recent) drinking occasion; ***High amount (number of drinks) of alcohol consumed during the last (most recent) drinking occasion.

The prevalence of a high length of time of drinking was most at Swansea (44%) and lowest at Oxford Brookes (25.1%), while the prevalence of high amount of drinking was most among Gloucestershire students (54.4%) and lowest at Oxford Brookes (26.8%). Prevalence of high frequency of drinking (drinking a few times or more each week) was again highest among Gloucestershire students (56.6%) and lowest in Ulster (27.7%). As regards frequency of heavy episodic drinking (consumed at least once ≥ 5 drinks in a sitting during last two weeks), the highest prevalence (74.7%) was that of Gloucestershire students, but this consumption pattern was lowest at Oxford Brookes (47.9%). Problem drinking (≥ 2 positive responses in CAGE) was reported by 28.8% of Ulster students, but only by 13.5% at Swansea. Possible alcohol dependence (≥ 3 positive responses in CAGE) was most often reported at Bath Spa (11.4%) and least often at Swansea (5.2%).

### Prevalence of six alcohol consumption indicators

Table 2 shows the prevalence of alcohol consumption indicators with respect to the variables under examination. The findings suggest that the prevalent alcohol use pattern among females was heavy episodic drinking, followed by large amount of drinking, high frequency of drinking, long duration of drinking, problem drinking and possible dependence. The same pattern (albeit at different rates) was also true for male students, where the most prevalent alcohol use pattern was heavy episodic drinking, followed by large amount of drinking, high frequency of drinking, long duration of drinking, problem drinking and possible dependence.

**Table 2:** Students’ sociodemographic and academic characteristics by six alcohol consumption indicators in the United Kingdom (Academic year 2007–2008)

	A. Long duration of drinking*	B. Large amount of drinking**	**C. High frequency of drinking**	**D. Heavy episodic drinking**	**E. Problem drinking**	**F. Possible alcohol dependence**
	**N (%)**	**N (%)**	**N (%)**	**N (%)**	**N (%)**	**N (%)**
**Gender**						
Female	857 (34.4)	1027 (41.7)	997 (37.7)	1521 (64.6)	496 (20.4)	167 (8.1)
Male	347 (48.6)	438 (62.3)	453 (60.2)	528 (76.4)	195 (29.3)	105 (15.8)
**Year of study**						
1st year	539 (39.2)	651 (48.4)	639 (44.2)	886 (68.7)	276 (20.4)	110 (8.1)
2nd year	412 (40.8)	488 (49.0)	440 (40.9)	682 (70.7)	236 (24.4)	94 (9.7)
3rd year	219 (35.7)	200 (45.2)	261 (40.7)	378 (64.3)	137 (24.6)	46 (8.3)
≥ 4th year	67 (27.5)	71 (29.6)	130 (50.2)	127 (54.7)	54 (22.9)	24 (10.2)
**Have intimate partner**						
Yes	633 (38.4)	777 (48.1)	664 (38.7)	1069 (68.1)	312 (19.9)	126 (8.6)
No	518 (41.0)	607 (48.6)	654 (48.3)	854 (71.6)	319 (25.4)	131 (10.6)
**Accomodation during semester**						
With parents	348 (42.3)	402 (49.4)	259 (29.6)	515 (64.6)	150 (18.5)	66 (8.1)
Other accomodation	923 (36.5)	1133(45.5)	1249 (47.0)	1621 (68.1)	564 (23.8)	213 (9.6)
**Parental education**						
Both parents high	850 (37.5)	1018 (45.6)	1008 (41.8)	1448 (66.4)	472 (21.4)	171 (7.7)
Mother high, father low	74 (37.9)	98 (50.3)	87 (42.9)	128 (68.4)	57 (30.5)	30 (16.0)
Father high, mother low	97 (36.2)	124 (47.5)	140 (51.5)	183 (72.0)	56 (22.9)	27 (11.0)
Both parents low	69 (40.8)	81 (49.1)	84 (46.7)	104 (66.7)	42 (27.1)	16 (10.3)
**Perceived income sufficiency**						
Always sufficient	454 (34.6)	548 (42.4)	608 (43.5)	800 (63.6)	252 (19.7)	103 (8.1)
Other	729 (40.5)	886 (49.8)	794 (42.2)	1195 (69.8)	409 (23.9)	160 (9.4)
**Educational (academic achievement) variables**						
**Important to achieve good grades**						
Higher importance	765 (37.5)	942 (46.7)	864 (40.0)	1296 (66.9)	426 (21.8)	160 (8.2)
Same/less importance	450 (38.2)	532 (45.9)	599 (47.8)	762 (67.4)	271 (23.4)	116 (10.0)
**Academic performance compared to peers**						
Same or better performance	969 (37.0)	1186 (46.0)	1185 (42.7)	1675 (67.0)	543 (21.5)	215 (8.5)
Lower performance	239 (41.0)	281 (49.0)	26.9 (43.9)	365 (67.3)	150 (26.7)	58 (10.3)

*Long duration (length of time) of last (most recent) drinking occasion; **Large amount (number of drinks) of alcohol consumed during the last (most recent) drinking occasion.

### Sociodemographic and academic characteristics associated with six alcohol consumption indicators

Gender was consistently related to all the six indicators of alcohol consumption that were examined. Female students were less likely to engage in long durations (length of time) of drinking, large amount of drinking, high frequency of drinking, heavy episodic drinking, and possible alcohol dependence. Chester and Swansea students were more likely to engage in long durations of drinking compared to the Bath Spa counterparts (Table 3, Section A); similarly Chester and Gloucestershire students reported significantly higher proportion of large amount of drinking during the last (most recent) drinking occasion. Conversely, Oxford Brookes students were significantly less engaged in large amounts of drinking when compared with Bath Spa. Gloucestershire students were more likely to engage in high frequency of drinking, while Ulster students were significantly less likely to engage in high frequency of drinking compared to Bath Spa. As for the frequency of heavy episodic drinking, Gloucestershire students reported significantly more heavy episodic drinking compared to Bath Spa (Table 3, Section D). Conversely, Oxford Brookes students were less likely to engage in heavy episodic drinking in comparison with those from Bath Spa (Table 3, Section D). In connection with problem drinking and possible alcohol dependence, Chester, Gloucestershire and Swansea students reported significantly less problem drinking and possible alcohol dependence in comparison with those from Bath Spa (Table 3, Sections E, F).

**Table 3:** Students’ sociodemographic and academic characteristics independently associated with six alcohol consumption indicators in the United Kingdom (Academic year 2007–2008)

	**A. Long duration of drinking**^***a***^	**B. Large amount of drinking**^***b***^	**C. High frequency of drinking**	**D. Heavy episodic drinking**	**E. Problem drinking**	**F. Possible alcohol dependence**
	**OR (95% CI)**	**OR (95% CI)**	**OR (95% CI)**	**OR (95% CI)**	**OR (95% CI)**	**OR (95% CI)**
**Gender**						
Female	.52 (.42–.64)***	.41 (.32–.52)***	.42 (.35–.56)***	.49 (.37–.66)***	.59 (.46–.75)***	.41 (.29–.57)***
Male	1	1	1	1	1	1
**University**						
Chester	1.39 (1.04–1.85)*	1.42 (1.07–1.88)*	.78 (.59–1.03)	1.03 (.75–1.40)	.67 (.48–.94)*	.51 (.31–.82)**
Gloucestershire	1.15 (.87–1.52)	1.61 (1.22–2.13)***	1.36 (1.04–1.79)*	1.36 (1.00–1.86)*	.66 (.48–.90)**	.57 (.37–.88)*
Ulster	.84 (.60–1.18)	1.15 (.83–1.59)	.54 (.39–.76)***	1.13 (.79–1.61)	1.14 (.79–1.65)	1.00 (.60–1.67)
Swansea	1.56 (1.13–2.16)**	1.35 (.98–1.87)	.77 (.56–1.06)	.95 (.67–1.33)	.46 (.31–.70)***	.33 (.17–.64)***
Plymouth	.86 (.56–1.32)	.77 (.50–1.17)	.66 (.43–1.00)	.68 (.44–1.05)	.83 (.45–1.51)	.58 (.23–1.44)
Oxford Brookes	.69 (.42–1.13)	.60 (.37–.97)*	.79 (.51–1.22)	.47 (.29–.74)***	.67 (.39–1.13)	.67 (.32–1.40)
Bath Spa	1	1	1	1	1	1
**Year of study**						
1st year	1.33 (.89–2.00)	1.63 (1.09–2.45)*	.99 (.67–1.47)	1.22 (.79–1.87)	.93 (.58–1.49)	.66 (.35–1.23)
2nd year	1.57 (1.04–2.36)*	1.71 (1.13–2.57)**	.85 (.57–1.26)	1.28 (.83–1.97)	1.05 (.66–1.69)	.72 (.39–1.34)
3rd year	1.18 (.76–1.82)	1.41 (.92–2.18)	.74 (.48–1.12)	.94 (.59–1.49)	1.01 (.61–1.68)	.55 (.27–1.09)
≥ 4th year	1	1	1	1	1	1
**Have intimate partner**						
Yes	.93 (.79–1.11)	.99 (.83–1.18)	.76 (.64–.89)***	.84 (.69–1.02)	.74 (.61–.91)**	.70 (.52–.94)*
No	1	1	1	1	1	1
**Accomodation during semester**						
With parents	1.21 (.99–1.46)	.68 (.44–1.05)	.43 (.29–.65)***	.39 (.24–.62)***	.67 (.52–.85)***	.83 (.59–1.18)
Other accomodation	1	1	1	1	1	1
**Parental education**						
Both parents high	.85 (.59–1.21)	.87 (.60–1.25)	1.01 (.69–1.45)	1.05 (.69–1.57)	.77 (.51–1.18)	.89 (.48–1.65)
Mother high, father low	.79 (.49–1.29)	.82 (.51–1.33)	1.16 (.71–1.89)	1.04 (.61–1.77)	1.20 (.70–2.06)	1.89 (.89–3.99)
Father high, mother low	.76 (.48–1.19)	.84 (.54–1.33)	1.23 (.78–1.94)	1.24 (.75–2.07)	.87 (.51–1.46)	1.18 (.55–2.51)
Both parents low	1	1	1	1	1	1
**Perceived income sufficiency**						
Always sufficient	.77 (.65–.92)**	.68 (.57–.81)***	.78 (.66–.93)**	.67 (.56–.82)***	.76 (.62–.94)*	.73 (.53–.99)*
Other	1	1	1	1	1	1
**Important to achieve good grades**						
Higher importance	.95 (.79–1.14)	.96 (.79–1.15)	.76 (.64–.91)**	.87 (.71–1.06)	.98 (.79–1.21)	.88 (.65–1.19)
Other	1	1	1	1	1	1
**Academic performance compared to peers**						
Same/better performance	.92 (.74–1.15)	.92 (.74–1.15)	.86 (.69–1.08)	1.03 (.81–1.32)	.74 (.57–.95)*	.68 (.48–.97)*
Lower performance	1	1	1	1	1	1
**Gender differences**						
Living with parents*Female	-	1.78 (1.10–2.89)*	–	2.13 (1.27–3.56)**	-	-

Findings from logistic regression models predicting the type of drinking, adjusted for all variables in the table; OR – odds ratio; ^*a*^Long duration (length of time) of last (most recent) drinking occasion; ^*b*^Large amount (number of drinks) of alcohol consumed during the last (most recent) drinking occasion; * *p* < 0.05; ** *p* < 0.01; *** *p* < 0.001.

According to year of study, 2^nd^ study year students were more engaged in long durations drinking, and 1^st^ and 2^nd^ year students were more engaged in large amount of drinking during the last (most recent) drinking occasion compared to their counterparts from the highest study year, but no associations were found between year of study and other alcohol indicators (Table 3). Being in an intimate partnership was negatively associated with high frequency of drinking, problem drinking and possible alcohol dependence, but not with long duration of drinking and large amount of drinking. In addition, accommodation with the parents during the semester was negatively associated with high frequency of drinking, heavy episodic drinking, and problem drinking among students, but not with long duration of drinking and large amount of drinking.

As regards socio economic status, there were no significant relationships between any of the six alcohol consumption variables and various combinations of parental (father’s and mother’s) educational status. However, perceived income insufficiency was significantly associated with all alcohol consumption indicators.

With respect to the two educational variables under examination, higher importance of achieving good grades was negatively associated with high frequency of drinking; and same or better academic performance compared to peers was negatively associated with problem drinking and possible alcohol dependence. However no associations between the educational variables and other alcohol consumption indicators were noted.

### Do associations between students’ sociodemographic and academic characteristics and the six alcohol consumption indicators differ by gender?

Finally, the two-way gender interactions showed that out of five potential interactions, two showed significant results. The associations between large amount of drinking, heavy episodic drinking and living with parents during the semester differed by gender, where there were more pronounced effects for female students (Table 3).

## Discussion

This research examined the alcohol consumption patterns of university students enrolled at seven universities in the UK, and investigated the associations between such drinking patterns and a range of sociodemographic and educational characteristics of students.

As for the first objective, the current study assessed the prevalence of alcohol use and found an overall high level of alcohol consumption. For example, a majority (59%) of students across our sample reported heavy episodic drinking within the last two weeks. Others [5] have also reported a higher level (67.2%) of heavy episodic drinkers in a Slovakian undergraduate student sample (77% of males, 51% of females). Conversely, in New Zealand [3], 37% of a total of 2,548 undergraduate students reported one or more binge episodes in the last week. In addition, 43% of our sample indicated drinking alcohol ≥ 2 times a week. Using a similar question to assess the frequency alcohol consumption among students from 7 universities across Europe, researchers [8] found that only Spanish students had a similar proportion (40%) of high frequency consumption, while the percentages were lower for students from Germany, Poland, Bulgaria, Denmark, Lithuania and Turkey [8]. As for the proportion of students with problem drinking (as indicated by CAGE), the UK sample was average (22% problem drinkers) when compared to other European university student samples where the prevalence of problem drinkers was between 16%-27% [8].

In relation to the study’s second objective, we examined the sociodemographic variables associated with six indicators of alcohol use. Out of all the variables that were scrutinized, male gender and insufficient income were the only variables consistently associated with all six indicators of alcohol consumption. Considerable research among European university students e.g. [7-9], and among students elsewhere [4,6] is in agreement with our overall finding of higher frequency of drinking, higher amount of heavy episodic drinking and higher amount of problem drinking among male students as compared to their female peers.

The finding of the current study that students who perceived their income as insufficient were more likely to drink and to be identified as problem drinkers is partly supported by other research [8] of students from 7 European countries. In that cross-European study [8], income insufficiency was associated with higher frequency of drinking, but not with problem drinking as measured by CAGE. However, due to the cross-sectional design, the directionality of effects remains unclear: whether the higher consumption of alcohol had contributed to financial problems and therewith to the perceived insufficient income; or if alcohol drinking is employed to cope with low income.

In relation to year of study, we found that students in the first two years of study were more likely to report large amount of drinking. This is in partial agreement with others, where in Slovakia, a higher study year was associated only with lower levels of heavy episodic drinking, but displayed no association with frequency of alcohol use, frequency of drunkenness and problem drinking [5]. However our findings are in contrast to research in Nigeria across 443 undergraduate university students, where older age was associated with problem drinking [4].

Regarding intimate partner relationships, our UK sample indicated that being in an intimate partnership was negatively associated with high frequency of drinking, problem drinking and possible alcohol dependence. We are in agreement with a review that found that university students without family obligations were more likely to consume alcohol in higher quantities [17]. Our findings are also in support of research of 813 university students in Slovakia [5], where having an intimate relationship was associated with less problem drinking only. However, the Slovakian study [5] did not measure possible alcohol dependence.

As for accommodation, in the current UK sample, accommodation with the parents during the semester was negatively associated with high frequency of drinking, heavy episodic drinking and also negatively associated with problem drinking among both genders. This is in agreement with research in Spain (750 students, ≤ 22 years), where living with friends was a risk factor for the consumption of alcohol, tobacco and illegal drugs, when compared with living at home [34]. Our sample’s findings are also in support of research of young adults across four universities in the Slovak Republic, where living with parents during the semester was consistently associated with less frequent heavy episodic drinking, drunkenness episodes, and problem drinking [5]. In Spain, research across university students suggested that, to decrease consumption among these young adults, strategies should target students who are living away from home [35]. In agreement, a recent review of 65 relevant articles published within the last 20 years reported that university students living alone, with roommates or in areas with a high density of students were more likely to consume alcohol in higher quantities [17]. However, as highlighted regarding sororities and fraternities in the USA, students who choose this type of residence exhibited a greater tendency towards substance consumption before joining [36]. Hence it still remains to be understood whether sharing accommodation is a risk factor for substance abuse or whether those who choose to live at home with their parents do so for reasons that also limit their contact with drugs [34].

Some of the indicators of alcohol consumption exhibited an inverse relationship with indicators of academic achievement. Students who felt that it is important for them to achieve good grades at university were less likely to report high frequency of drinking. Likewise, students who rated their academic performance as equal or better than that of their peers were less likely to exhibit problem drinking or suspicion of alcohol dependence. Our findings are in accordance with other studies showing that alcohol consumption has been negatively associated with academic performance [18], or to missed classes and poor academic achievement [19].

In connection to the third objective of the study, we found only limited evidence that the associations between students’ characteristics and alcohol consumption indicators differed by gender. We found significant associations only for the variable 'living with parents’. For female students, living with parents had a stronger negative effect on large amount of drinking and on heavy episodic drinking than for male students. Since all other associations did not show any gender effect, the current finding suggested that alcohol drinking is influenced by quite similar determinants for both female and male students.

In addition to investigating the factors associated with alcohol consumption above, the study contributed to existing research on alcohol use in university students in several ways: Firstly, due to the fact that we included six indicators of alcohol consumption, we were able to show that students had high prevalences (females more than 30%, and males about or more than 50%) across all indicators measuring frequency and high quantity of use. Since the CAGE test was the only measure that indicated a lower prevalence of alcohol related problems, one might come to the conclusion that students’ drinking style could be transient and may not cause serious problems. However, one might also argue that the CAGE instrument is more likely to show the effects of year long high alcohol intake and could therefore be less adequate to be used in young populations or to demonstrate short-term consequences of alcohol misuse. Secondly, the study showed that the amount and frequency of drinking differed substantially between universities even when adjusted for student composition. Therefore studies using student samples from just one university may come to misleading prevalence estimates. It was also interesting to note that some universities had higher prevalence of the amount and frequency of drinking while they had a lower prevalence of problem drinking. This might indicate differences in the drinking culture/s and local norms, and underlying institutional factors such as differences in alcohol policies that could contribute to such findings.

This study has limitations and generalizations of the findings should be cautious. Data was self-reported and potential recall bias and social desirability/sociability might play a role. Participants were recruited at universities and those absent, possibly due to health reasons or even due to problem drinking, might not have had alternative opportunity to participate. Students less/un interested in healthy practices might have been less apt to participate and might therefore be under-represented in our samples. As a general student health survey undertaken within the lectures, due to the time limitations and in order to minimize respondent burden, some features were assessed by single item measures. For the same reason some other indicators of alcohol use, e.g. total number of drinks consumed per week were not included in the questionnaire. At each participating university, we widened the data collection in order get student samples that were representative of their universities; however, this UK sample remains a convenience sample. Such convenience samples are common in general health and wellbeing surveys of university/college students, usually due to the research interests of and past collaborations between the participating institutions. A detailed comparison of potential explanations of the differences found between the participating universities would have been possible if the collected data would have included variables related to the background of the University, background of students and the wider situational context. Future research should attempt to address these limitations.

## Conclusion

The current study found a high level of alcohol consumption among students from different universities in the UK, which calls for more regular/periodic assessments of student’s health and wellbeing, and also for preventive action at universities across the UK. Several socio-demographic variables were associated with the different indicators of alcohol consumption. The findings can inform prevention strategies at universities in order to allocate interventions to student groups with the highest likelihood of heavy drinking and drinking problems such as male students, students living outside parents’ homes (e.g. in dorms) and those in lower years of their studies. Effective programs should educate student groups about the responsible use of alcohol, and to also to inform student groups about their potential susceptibilities to alcohol misuse and possible dependency. Beyond educational programs, measures and policies aiming at limiting the availability of alcohol would be necessary in order to achieve a sustainable reduction in hazardous alcohol consumption among students.

## Competing interests

The authors declare that they have no competing interest.

## Authors’ contributions

The authors of this manuscript discussed the topic for considerable time. WEA conceived the study and drafted the first version of the manuscript. RS undertook the statistical analysis, RS and CS revised the manuscript and gave consent for the version to be published. All authors read and approved the final manuscript.
